# Smart Tactile Sensing Systems Based on Embedded CNN Implementations

**DOI:** 10.3390/mi11010103

**Published:** 2020-01-18

**Authors:** Mohamad Alameh, Yahya Abbass, Ali Ibrahim, Maurizio Valle

**Affiliations:** 1 Department of Electrical, Electronic and Telecommunication Engineering and Naval Architecture (DITEN)-University of Genoa, via Opera Pia 11a, 16145 Genova, Italy; Mohamad.Alameh@edu.unige.it (M.A.); Yahya.Abbass@edu.unige.it (Y.A.); Maurizio.Valle@unige.it (M.V.); 2 Department of Electrical and Electronics Engineering, Lebanese International University (LIU), Beirut 1105, Lebanon

**Keywords:** tactile sensing systems, embedding intelligence, convolutional neural network

## Abstract

Embedding machine learning methods into the data decoding units may enable the extraction of complex information making the tactile sensing systems intelligent. This paper presents and compares the implementations of a convolutional neural network model for tactile data decoding on various hardware platforms. Experimental results show comparable classification accuracy of 90.88% for Model 3, overcoming similar state-of-the-art solutions in terms of time inference. The proposed implementation achieves a time inference of 1.2 ms while consuming around 900 μJ. Such an embedded implementation of intelligent tactile data decoding algorithms enables tactile sensing systems in different application domains such as robotics and prosthetic devices.

## 1. Introduction

**Figure 1:**
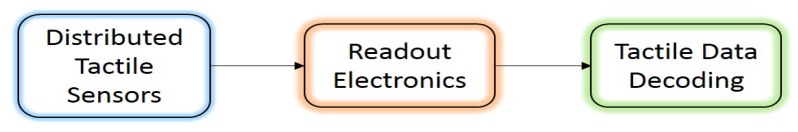
Block diagram of the tactile sensing system.

Embedding intelligence near the sensor location may enable tactile sensing systems to be incorporated in many application domains such as prosthetics, robotics, and the Internet of Things. Tactile sensing systems are composed of three main parts, as shown in Figure 1. The distributed tactile sensors are in charge of converting the mechanical stimuli applied on their surface into electrical signals. Tactile sensors could be made from different materials, e.g., capacitive, piezoelectric, and piezoresistive materials [1]; they should be able to enable capabilities similar to what happens on the human skin such as normal and shear force detection, vibration detection, softness, texture, shapes, etc. The readout electronics interface with the sensor arrays by acquiring and digitizing the electrical signals to be then processed by the digital tactile data processing unit [2].

Decoding tactile information concerns different kinds of tasks, which could be categorized as: simple or complex processing depending on the algorithm’s complexity. For simple processing, an example of the information retrieved is temperature, the intensity of the contact force, and contact location, direction, and distribution. Concerning complex processing, more intelligent tasks are expected such as patterns, textures, and roughness classification or touch modalities’ discrimination. Employing the complex processing approach enables intelligence in tactile sensing systems. It is achieved by applying sophisticated and complex data decoding algorithms able to extract the meaningful information from sensors. Machine learning (ML) has emerged as an efficient method in many fields and in everyday tasks in smartphones and electronic systems. ML is a powerful learning from examples paradigm used to address classification and regression problems. In particular, Convolutional (CNN) and Deep Neural Networks (DNN) have recently proven their effectiveness when applied to image recognition and tactile data decoding [3]. Many recent research works have focused on the development of ML algorithms for tactile sensing systems [4]. However, embedding machine learning algorithms on hardware platforms near the sensors location is challenging due to the complexity such algorithms impose in terms of time latency and energy consumption. Our main goal is to achieve a tactile sensing system able to perform smart tasks. This system is intended to be portable/wearable, for which the energy budget is limited. Moreover, for the target applications, i.e., robotics and prosthetics, being lightweight is a critical constraint limiting the hardware and battery size.

In this perspective, this paper presents the implementation of CNN algorithms on different hardware platforms. The main contribution of this paper may be summarized as follows:

- It proposes an optimized CNN model, adopted from Gandarias et al.’s research [5], based on reduced data, which demonstrates the ability to provide comparable results in terms of accuracy, i.e., 90.88%, with reduced hardware complexity.
- It presents efficient implementations of the CNN model on different hardware platforms for embedded tactile data processing. The proposed implementations achieve a time inference of 1.2 ms while consuming around 900 μJ. The work demonstrates its suitability for real-time embedded tactile sensing systems.
- It raises a discussion about integrating intelligence into tactile sensing systems and how it enables tactile sensing systems in different application domains.

The remainder of this paper is organized as follows: Section 2 reports the state-of-the-art, showing the recent embedded CNN implementations. In Section 3, we illustrate the experimental setup and methodology. In Section 4, the hardware implementation is explained. The results and discussion are presented in Section 5, followed by the conclusions in Section 6.

## 2. State-of-the-Art

Different works have addressed the tactile data classification problem, using different methods including, but not limited to, machine learning and deep learning [6,7,8,9,10]. While most of the work done was focused on the methodology itself, few works addressed the implementation on embedded platforms where the real application should reside. Gandarias et al. [11] used two approaches to classify eight objects: finger, hand, arm, pen, scissors, pliers, sticky tape, and Allen key, using a 28 × 50 tactile sensory array attached to a robotic arm, the first approach using the Speeded-Up Robust Features (SURF) descriptor, while the second a pre-trained AlexNet CNN for feature extraction, with a Support Vector Machine (SVM) classifier for both approaches. In Yuan et al.’s research [12], a CNN was also used for active tactile clothing perception, to classify clothes grasped by a robotic arm equipped with a tactile sensor that output a large RGB pressure map. Based on different textile properties: thickness, smoothness, textile type, washing method, softness, stretchiness, durability, woolen, and wind proof. Each property held two or more labels, e.g., the thickness can be a number from 0–4, and the employed model for textile classification was VGG-19 pretrained on ImageNet [13]. In Rouhafzay et al. [14], a combination of virtual tactile sensors and visual guidance was employed to distinguish eight classes of simulated objects; the tactile sensor size was 32 × 32, and the input size of the neural network was 32 × 32 × 32, which was a sequence of tactile sensor images. Abderrahmane et al. [15] introduced a zero-shot object recognition framework, to identify previously unknown objects based on haptic feedback. They used BioTac sensors, and two CNNs were employed: one for visual data (input size: 224 × 224 × 30) and the other for tactile data (32 × 30). They overcame the results of SVM in a previous work [16]. In Alameh et al.’s research [3], transfer learning was used to classify touch modalities obtained through a small 4 × 4 piezoresistive sensory array, by transforming tensorial data into images and then using different CNN models trained on ImageNet [13]. In Gandarias et al.’s research [5], they used a light CNN based (only three convolutional layers inside) on AlexNet, to identify 22 objects using their pressure map, collected from a 28 × 50 tactile sensory array. Other works include those in [17,18,19].

While all these previous works were not implemented in an embedded environment, we can find few others targeting an embedded implementation for tactile sensing applications. The need for embedded implementation arises from the need to have low power, small form factor electronics to process the tactile information, especially in prosthetic applications [20]. Osta et al. [21] demonstrated an energy efficient system for binary touch modality classification, based on SVM and implemented on a custom hardware architecture. The energy per inference was 81 mJ, and the inference time was 3.3 s. Ibrahim et al. [22] presented a real-time implementation on FPGA for touch modality classification. Using SVM, they achieved a 350 ms inference time and a 945 mJ inference energy for three class classification, as well as 970 ms/6.01 J for a five class classification.

## 3. Experimental Setup and Methodology

### 3.1. Dataset

**Figure 2:**
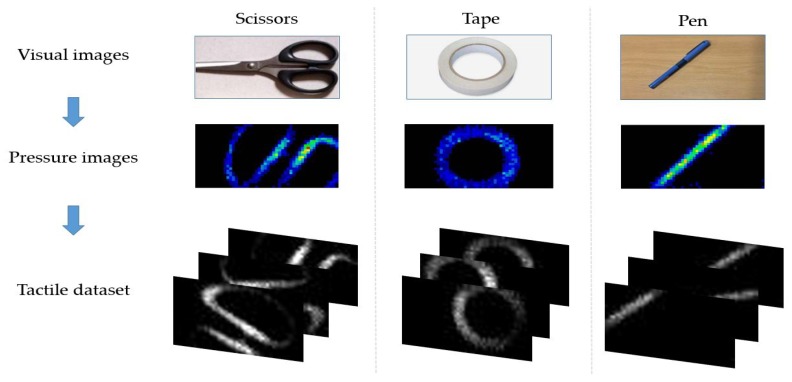
Examples of visual (**top**) vs. pressure (**middle**) vs. tactile images (**bottom**) of common objects.

Targeting the classification of tactile data, the use of the dataset collected in [5] was considered. Tactile data were collected by a high resolution (1400 pressure taxels) tactile array, which was attached to the 6 DOF robotic arm AUBO Our-i5 [5]. A set of piezoresistive tactile sensors was distributed with a density of 27.6 taxels/cm${}^{2}$, forming a matrix of 28 rows by 50 columns. The dataset was composed of pressure images that presented the compliance of 22 objects with the tactile sensors. These images were divided into 22 classes labeled as adhesive, Allen key, arm, ball, bottle, box, branch, cable, cable pipe, caliper, can, finger, hand, highlighter pen, key, pen, pliers, rock, rubber, scissors, sticky tape, and tube. Figure 2 shows an example of the tactile images of three objects used for the training of the CNN model. Each taxel in the tactile array presents a pixel in the pressure image; thus, each pressure image is 28 × 50 × 3 in size. Therefore, the color of the pixel presents the pressure applied at the corresponding taxel. The minimum pressure is presented by black color, and the maximum pressure is presented by red color. Pressure images were then transformed into grayscale images (image size = 28 × 50 × 1), forming the tactile dataset.

### 3.2. Tested Model

**Figure 3:**
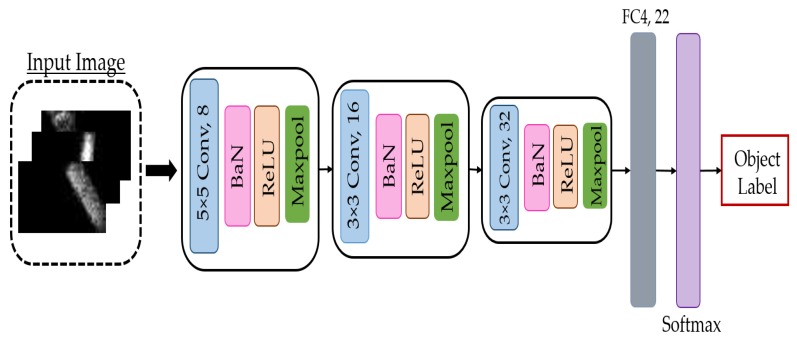
Architecture of the tested model. BaN, Batch Normalization.

Due to computational and memory limitations in the embedded application, a light CNN model was required to perform classification tasks with high accuracy and fewer parameters. In this work, we chose to use one of the models implemented in [5] as a base model to classify the objects in the aforementioned dataset. Among all the implemented networks, we chose to use the custom network TacNet4 because it was the best network that fit the embedded application (fewer parameters with high accuracy [5]). The model was based on AlexNet, which is usually used in computer vision for object classification [23]. The network was composed of 3 Convolutional layers (Conv1, Conv2, and Conv3) with filters sizes (5 × 5, 8), (3 × 3, 16), and (3 × 3, 32) respectively. Each convolutional layer was followed by a Batch Normalization (BaN), Activation (ReLU), and Maxpooling (Maxpool) layer, respectively, where all pooling layers used 2 × 2 maxpooling with a stride of two. A Fully Connected layer (FC = [fc4]) with 22 neurons followed by a softmax layer were used to classify the input tactile data and give the likelihood of belonging to each class (object). The input shape of the model was configured to the size of the collected tactile data. Figure 3 shows the detailed structure of the network used.

The network was implemented in MATLAB R2019b using the Neural Network Toolbox. A total of 1100 tactile images were used to train the model. The learning process was implemented in MATLAB by dividing the tactile data into three sets: training, validation, and test sets.

When having an adequate dataset, the validation set is expected to be a good statistical representation of the entire dataset. If not, the results of the training procedure highly depend on how the dataset is divided.

**Figure 4:**
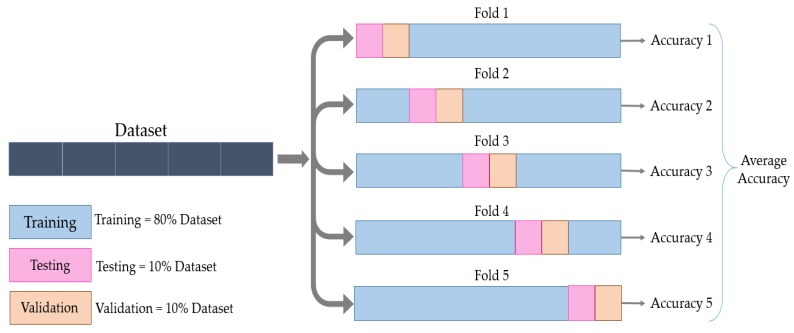
Visual representation of the training, test, and validation split using cross-validation.

To avoid this, In this work, we used the cross-validation method. The data were partitioned into five folds, and each fold was divided into training, validation, and test sets. The training set formed 80% of the dataset, and the validation and test sets formed 10% each. This process was then repeated five times until all the folds were used, without having common elements across all folds for the validation and test sets, as shown in Figure 4.

For each training process, the training set was composed of 880 images, 40 images for each label, whilst each of the validation and test sets was composed of 110 images. Training the model from scratch required a large dataset to achieve high accuracy. For that reason, data augmentation techniques, i.e., flipping, rotation, and translation in the *X* and *Y* axis, were applied to the dataset. Hence, the amount of tactile data available for training and validation was increased to 5280 and 660, respectively. The performance of the implemented model was evaluated based on the recognition rates achieved in a classification experiment of the test set composed of 110 original images (objects) from 22 classes.

**Figure 5:**
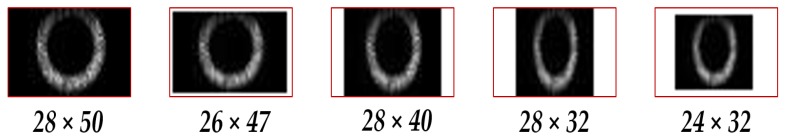
Example of an image resized for the sticky tape object; the red canvas is shown for illustration, which signifies the original image size (28 × 50).

For embedded applications, with computational, memory, and energy constraints, it is necessary to decrease the number of trainable parameters in the CNN model. In this work, we chose to decrease the number of parameters of the trained model by decreasing the input image size (i.e., lower resolution images); an example is shown in Figure 5. For that reason, several experiments were performed to choose the smaller size of the input data, keeping the same classification accuracy. The input shapes were chosen in a way that each shape resulted in a reduction of the number of parameters.

**Figure 6:**
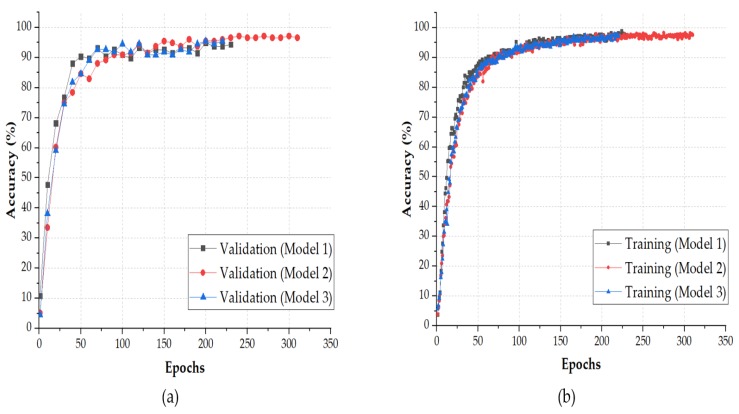
Learning accuracy for the 3 configurations of the TactNet4model: (**a**) training; (**b**) validation.

Table 1 shows how the number of parameters of the layers depended on the input shape. The change in the input shape affected only the number of parameters of the fully connected layer. This was due to the fact that the number of parameters in the convolutional layer depended only on the size and number of the filters assigned for each layer (((width of the filter × height of the filter) + 1) × No. of filters), while in the FC layer, the number of parameters ((No. of neurons in the FC layer × No. of neurons in the previous layer) + 1) was affected by the size of the input image and the output layer. The performance of the model was studied with five different input shapes, as shown in Table 1. This resulted in five different models with different input shapes, each one trained from scratch 5 times (one time per fold), which output 25 trained NNs. Figure 6 shows the training and validation accuracy over epochs, for the first three models among the five models. The figure shows that the accuracy achieved by the three models was close to 100%. Each model was evaluated with MATLAB by running a classification task on the test set.

**Figure 7:**
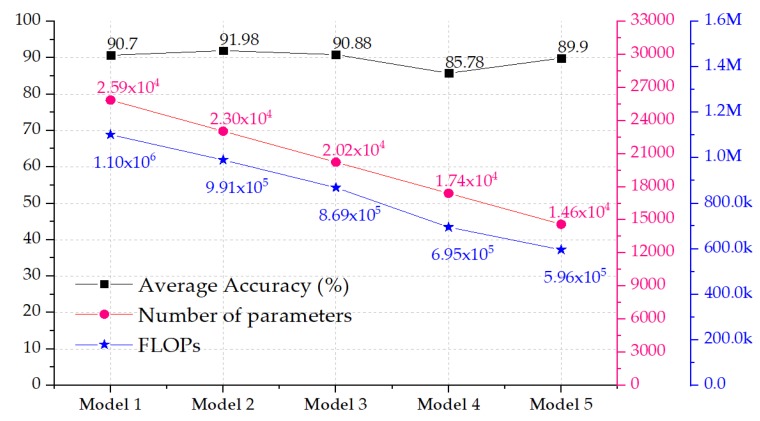
Comparison of the performance, number of trainable parameters, and FLOPS in the convolutional layers.

Figure 7 shows the change in the number of trainable parameters and the average classification accuracy, with respect to the change in the input shape, as well as the FLOPs. The classification accuracy presented the average test accuracy among the five folds. The figure shows that it was possible to decrease the input size from 28 × 50 × 1 to 26 × 47 × 1 or to 28 × 40 × 1 and achieve an increase in the classification accuracy from 90.70% to 91.98% and 90.88%, respectively. Decreasing the input size of the model resulted in a drop in the trainable parameters from 25,862 to 23,046 and 20,230 parameters, respectively, for the aforementioned models. This decrease in the number of parameters would also induce a decrease of the number of Floating Point Operations (FLOPs), as shown in Figure 7; the average ratio of the decrease in the number of parameters with respect to the decrease in the number of FLOPs was 1/44 i.e., with each decrease in number of parameters, there was a 44 times decrease of the FLOPs. The number of FLOPS in Figure 7 corresponds to the convolutional layers only, where most of the FLOPs were, and these FLOPs were calculated according to the following formula [24]: FLOPs = n × m × k, where n is the number of kernels, k is the size of the kernel (width × height × depth), and m the size of output feature map (width × height), while the depth in the kernel size corresponds to the depth of the input feature map.

## 4. Embedded Hardware Implementations

**Figure 8:**
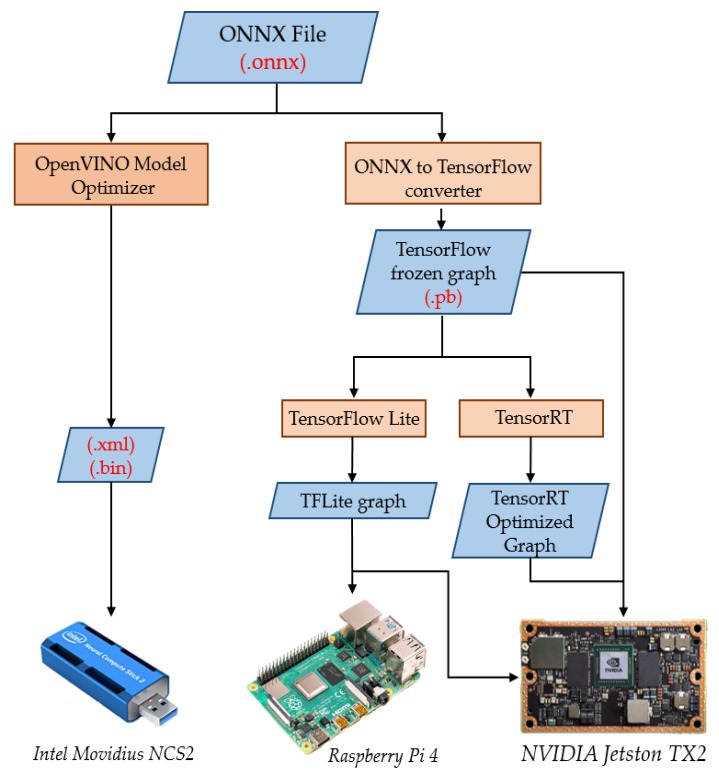
Implementation flow.

The models obtained from MATLAB were converted to Open Neural Network Exchange (ONNX) format [25]. ONNX provides an open source format for AI models, both deep learning and traditional ML, which enables the inter-operability between different frameworks. Figure 8 shows how the CNN model was converted into different formats for different hardware platforms. Figure 7 shows the number of trainable parameters and the corresponding accuracy for each model. It is clearly shown that all models preserved comparable accuracy, but the best were the first three, i.e., Model 1, Model 2, and Model 3. However, since Model 2 and Model 3 demonstrated a reduced number of training parameters and accordingly a reduced number of operations (FLOPS), they were selected for the hardware implementation. This choice was based on the fact that reducing FLOPS reduced the inference time and power consumption.

The reason behind the selection of hardware platforms was as follows:

1. The custom architecture targeting the embedded implementation of neural networks, e.g., Movidius NCS2.
2. The high usability of ARM processors in embedded architectures, e.g., Raspberry Pi 4.
3. The high performance architecture, designed for parallel processing in general, and also optimized for embedded applications: e.g., NVidia Jetson TX2.
4. The support for the execution of pretrained neural network models coming from different platforms without retraining.

### 4.1. Movidius Neural Compute Stick 2

Movidius Neural Compute Stick 2 (NCS2) is a hardware accelerator designed by Intel for on-chip neural network inference, especially CNNs, equipped with the Intel Movidius MyriadX Vision Processing Unit (VPU). It contains 16 SHAVE (Streaming Hybrid Architecture Vector Engine) cores [26] and a dedicated hardware neural network accelerator. It requires a host to flash the neural network, as well as to feed it with data and invoke the inference to get the results back via the USB 3.0 port. The host can be a Linux, Windows, or Mac based machine. To achieve these tasks, Intel provides OpenVINO: Open Visual Inference and Neural network Optimization Toolkit, a cross platform toolkit that enables deep learning inference and easy heterogeneous execution across multiple Intel® hardware (VPU, GPU, CPU, FPGA). The optimizations offered by OpenVINO are: batch normalization and scale shift, linear operation merge and linear operation fusion. The details were mentioned in [27].

### 4.2. Jetson TX2

NVidia’s Jetson TX2 [28] is a power efficient embedded AI computing device, designed mainly for edge AI, and belongs to the Pascal™ family of GPUs, loaded with 8 GB of memory, 59.7 GB/s of memory bandwidth, and 8 GB of RAM. In this experiment, we used TensorFlow (TF) [29] for the inference, as well as NVidia TensorRT [30] under Ubuntu OS. TF is an open source end-to-end machine learning platform, while TensorRT is a platform for high performance deep learning inference dedicated to NVidia hardware. It includes a deep learning inference optimizer and a runtime that delivers low latency and high throughput for deep learning inference applications.

As an optimization for TensorFlow, TensorFlow Lite (TFLite) [31] is an open source deep learning framework for on device inference. The same TensorFlow model can be converted into the TFLite model. To perform an inference with a TFLite model, the TFLite interpreter is required, which uses a static graph ordering and a custom (less dynamic) memory allocator to ensure minimal load, initialization, and execution latency [31], also reducing the weights’ precision, e.g., floating point vs. fixed point precision, without affecting the accuracy.

### 4.3. ARM

As for the implementation on the ARM architecture, we used Raspberry Pi 4, equipped with a Quad core Cortex-A72 (ARM v8) 64-bit System on Chip (SoC) @ 1.5 GHz and 4 GB RAM. For the inference on this hardware, we used the TFLite runtime library, under the Ubuntu OS.

For all the mentioned platforms, both power consumption and inference time were calculated. The inference time was calculated by averaging 110 inferences, which corresponded to the test set size. As for the power consumption, two methods were used:

1. the provided APIs in Jetson TX2, which provided readings about voltage, power, and input current to the GPU.
2. the external USB multimeter, connected in serial to the power source for both Raspberry Pi and the Movidius NCS2.

## 5. Results and Discussion

In this work, we achieved a better accuracy in tactile data classification using CNN compared to the original model obtained in [5], even by resizing the input, therefore decreasing the number of trainable parameters. The chosen models reduced the number of trainable parameters by a maximum of 21.77% of the original trainable parameters and also increased the accuracy by a maximum of 1.28%, noting that Model 5 (24 × 32 × 1) with 0.8% less accuracy than the original model had 42% fewer trainable parameters. Choosing the right model depended on the implementation, i.e., a trade-off between accuracy and hardware complexity should take place: if the best accuracy was targeted, then Model 2 should be selected; while the choice of Model 3 would be when less hardware complexity was needed, accepting a small accuracy degradation. Reducing the input size while still keeping the same or even better accuracy could be explained in three points:

1. The random initialization of the weights may lead in different runs to different accuracy results, e.g., 10 different runs for training Fold 4 of Model 2 with the same hyperparameters gave different results, as shown in Table 2, which shows an average of 94.36% and a standard deviation of 1.904%.
2. Random selection of batch data and data shuffling would affect also the update of the weights and make them different from one training to another.
3. The feature extraction process achieved by CNN was error resilient [32]. A CNN could still extract features even with some manipulation of the input image. This was one of the reasons for data augmentation [33] when training neural networks, which was to let the neural network learn the features even from augmented images (scaled, rotated, flipped, etc.), instead of learning only the samples in the original dataset. In our case, the features were still detectable even after image resizing, as shown in Figure 5.

According to Table 3 and Table 4, the smallest power consumption and inference time were obtained using TensorRT under Jetson TX2, which was 153 mW dynamic power within 5.29 ms as the inference time, implying 0.809 × 10 ${}^{-3}$ Joules of dynamic energy (see Table 5). The most dynamic energy consumption was for the Intel Movidius NCS2, 1.9 ms × 800 mW = 1.52 × 10 ${}^{-3}$ Joules. Regarding the power consumption, since the neural network used was small compared to the hardware capacity, the power consumption was almost the same for the three models, noting that the accuracy on the USB power meter was on the 10 mW scale, so that a difference of less than 10 mW between two measurements could not be detected using this instrument.

## 6. Conclusions

This paper presented the implementation of a smart tactile sensing system based on an embedded CNN approach. The proposed model optimized a state-of-the-art model proposed in [5] by reducing the input data size. The experimental results were comparable in terms of accuracy after reducing the size from (28 × 50) to (26 × 47) and (28 × 40). The hardware implementation on different hardware platforms, namely Movidius NCS2, NVidia’s Jetson TX2, and Cortex-A72 (ARM v8), was provided. The proposed models showed better performance on hardware platforms when time inference was compared. Power consumption was also measured and compared among different platforms. Targeting portable tactile sensing systems, the proposed work demonstrated the feasibility of integrating machine learning methods on a hardware platform to enable intelligence for such a system. This may pave the way for smart tactile sensing systems to be applied in prosthetics and robotics.

## Figures and Tables

**Table 1:** Distribution of the number of parameters on the models’ layers.

Layers	Model 1	Model 2	Model 3	Model 4	Model 5
	(28 × 50)	(26 × 47)	(28 × 40)	(28 × 32)	(24 × 32)
Conv1	208	208	208	208	208
BaN1	16	16	16	16	16
Conv2	1168	1168	1168	1168	1168
BaN2	32	32	32	32	32
Conv3	4640	4640	4640	4640	4640
BaN3	64	64	64	64	64
FC	19,734	16,918	14,102	11,286	8470
Total	25,862	23,046	20,230	17,414	14,598

**Table 2:** Accuracy results for 10 runs on Model 2, Fold 4.

Trials	Accuracy (%)
1	96.36
2	92.73
3	94.55
4	91.82
5	97.27
6	93.64
7	92.73
8	95.45
9	96.36
10	92.73
Average ± Stdev	94.36 ± 1.904%

**Table 3:** Comparison of the inference time between models.

Platform		Inference Time (ms)		
Hardware	Software	Model 1	Model 2	Model 3
Jetson TX2	TensorRT	5.5597	5.2905	5.919
	TF	6.2943	5.4691	5.946
	TFLite	1.3384	1.2181	1.2445
Core i7	MATLAB	3.245	2.6139	2.4715
Movidius NCS2	OpenVINO	1.9	1.9	1.86
Raspberry Pi4	TFLite	1.615	1.473	1.21

**Table 4:** Power consumption.

Platform		Current (mA)		Voltage (V)	Consumed Power (mW)		
Hardware	Software	Static	Total		Static	Total	Dynamic
Jetson	TensorRT	8	16	19.072	152	305	153
	TF	8	16	19.072	152	305	153
Movidius NCS2	OpenVINO	-	160	5	-	800	800
Raspberry Pi4	TFLite	560	700	5	2800	3500	700

**Table 5:** Energy consumption.

Platform		Energy Consumption (μJ)		
Hardware	Software	Model 1	Model 2	Model 3
Jetson TX2	TensorRT	850.6341	809.4465	905.607
	TF	963.0279	836.7723	909.738
Movidius NCS2	Open VINO	1520	1520	1488
Raspberry Pi4	TFLite	1130.5	1031.1	847

## Abbreviations

The following abbreviations are used in this manuscript:

CNN	Convolutional Neural Network
DCNN	Deep Convolutional Neural Network
SVM	Support Vector Machine
ML	Machine Learning
FPGA	Field-Pogrammable Gate Array
